# Is Patient‐Specific Instrumentation Accurate and Necessary for Open‐Wedge High Tibial Osteotomy? A Meta‐Analysis

**DOI:** 10.1111/os.13483

**Published:** 2022-12-30

**Authors:** Ran Pang, Zhaohui Jiang, Chunlei Xu, Wei Shi, Xinglong Zhang, Xin Wan, Daniel Bahat, Hui Li, Fedor Senatov, Inna Bulygina, Hu Wang, Huafeng Zhang, Zhijun Li

**Affiliations:** ^1^ Department of Orthopaedics Tianjin Medical University General Hospital Tianjin PR China; ^2^ Department of Orthopaedics Cleveland Clinic Cleveland Ohio USA; ^3^ Department of Orthopaedics Tianjin Hospital of ITCWM Nankai Hospital Tianjin PR China; ^4^ Center for Biomedical Engineering National University of Science and Technology “MISIS” Moscow Russia; ^5^ Department of Physical Health Care and Rehabilitation Tianjin Vocational College of Sports Tianjin PR China

**Keywords:** High tibial osteotomy, Meta‐analysis, Osteoarthritis, Patient‐specific cutting guides, Patient‐specific instrumentation, Three‐dimensional

## Abstract

The purpose of this meta‐analysis was to identify if patient‐specific instrumentation (PSI) could increase the accuracy of the correction in high tibial osteotomy (HTO) and to explore the assessment indices and the necessity of using a PSI in HTO. A systematic search was carried out using online databases. A total of 466 patients were included in 11 papers that matched the inclusion criteria. To evaluate the accuracy of PSI‐assisted HTO, the weight bearing line ratio (WBL%), hip‐knee‐ankle angle (HKA), mechanical medial proximal tibial angle (mMPTA), and posterior tibial slope angle (PTSA) were measured preoperatively and postoperatively and compared to the designed target values. Statistical analysis was performed after strict data extraction with Review Manager (version 5.4). Significant differences were detected in WBL% (MD = −36.41; 95% CI: −42.30 to −30.53; *p* < 0.00001), HKA (MD = −9.95; 95% CI: –11.65 to –8.25; *p* < 0.00001), and mMPTA (MD = –8.40; 95% CI:−10.27 to −6.53; *p* < 0.00001) but not in PTSA (MD = 0.34; 95% CI: −0.59 to 1.27; *p* = 0.47) between preoperative and postoperative measurements. There was no significant difference between the designed target values and the postoperative correction values of HKA (MD = 0.14; 95% CI: −0.19 to 0.47; *p* = 0.41) or mMPTA (MD = 0.11; 95% CI −0.34 to 0.55; *p* = 0.64). The data show that 3D‐based planning of PSI for HTO is both accurate and safe. WBL%, HKA, and mMPTA were the optimal evaluation indicators of coronal plane correction. Sagittal correction is best evaluated by the PTSA. The present study reports that PSI is accurate but not necessary in typical HTO.

## Introduction

In recent years, medial open‐wedge high tibial osteotomy (HTO) has typically been performed to treat medial interventricular osteoarthritis of the knee, medial compartment overload, and osteochondral lesions with varus deformities.^1^ Total knee arthroplasty (TKA) is an optimal treatment for patients with end‐stage osteoarthritis. For young and energetic patients, HTO is well recognized as a conservative surgical option that avoids the need for premature total knee arthroplasty.^2^ A series of studies have confirmed that HTO achieves good clinical results with strict adherence to operation indications and precise surgical techniques.^3^, ^4^ The latter depends on achieving accurate correction in all spatial planes (especially in the coronal and sagittal planes).^5^, ^6^, ^7^, ^8^ It is generally accepted that precise alignment is essential in achieving long‐term clinical outcomes.

To achieve the optimal planned correction, there are three methods of preoperative planning: conventional radiography, navigation, and three‐dimensional printed patient‐specific instrumentation (PSI). Navigation is also known as computer‐assisted surgery (CAS), and the PSI is called patient‐specific cutting guide (PSCG).^9^, ^10^ Many studies have compared navigation and conventional HTO.^11^ There is general agreement that it is difficult to achieve accurate preoperative correction with conventional radiography alone.^12^, ^13^, ^14^ According to some authors, CAS and PSI increase the precision of osteotomy. Other scholars believe that CAS cannot sufficiently control the degree of the desired correction.^8^ A recent meta‐analysis demonstrated that CAS and PSI achieve increased coronal alignment accuracy.^11^ However, the meta‐analysis mainly focused on the description of CAS. Additionally, the authors made no final conclusion comparing PSI to traditional surgical techniques.

To date, there are no RCTs assessing whether a PSI improves the accuracy of HTO and whether the application of a PSI in HTO results in better outcomes. The existing literature on PSIs consists of observational studies. These authors evaluated the accuracy, safety, and precision of the three methods. They demonstrated improved clinical outcomes directly related to refined revisions in the alignment of the lower limb using WBL% (weight bearing line ratio), mMPTA (mechanical medial proximal tibia angle), HKA angle (hip‐knee‐ankle angle), and PTSA (posterior tibial slope angle).^10^, ^15^, ^16^ However, there is no consensus in the current literature on which assessment indices yield optimal outcomes. In addition, the application of PSIs in HTO is also controversial, given the increased radiation exposure for doctors and patients, increased patient costs, and potential complications caused by a PSI. It is also worth considering whether a PSI is necessary for conventional HTO. As some scholars have proposed, PSIs are applicable in highly complex surgical interventions, especially those that require multiplanar or rotational osteotomies.^17^ Patients can often obtain satisfactory clinical outcomes. Unfortunately, there is a lack of objective data on the necessity of the application of PSIs in HTO.

The aims of this meta‐analysis were (i) to identify whether PSI could increase the accuracy of the correction in HTO and (ii) to explore the various assessment indices and the necessity of using PSI in HTO.

## Methods

This meta‐analysis was conducted in accordance with the recommendations proposed by the Preferred Reporting Items for Systematic Reviews and Meta‐analyses (PRISMA) statements.^18^

### Literature Search

According to the Cochrane Collaboration guidelines, systematic searches were performed to identify pertinent articles from online databases, including Embase, PubMed, and the Cochrane Central Register of Controlled Trials, using the search terms “medial open‐wedge high tibial osteotomy,” “patient‐specific cutting guides,” and “patient‐specific instrumentation.” These databases were searched from their creation dates through October 16, 2021. The search retrieved 162 results of possible relevance. Additionally, the references of the relevant studies were hand searched. Two reviewers (R. P. and Z. J.) independently assessed the included articles. Discrepancies were resolved by consensus.

### Inclusion and Exclusion Criteria

Articles with the following characteristics were included: (i) subjects undergoing open‐wedge HTO to treat medial interventricular osteoarthritis or varus deformity of the knee caused by any reason; (ii) subjects who underwent PSI surgery with preoperative planning by 3D technology; (iii) use of an evaluation index for HTO accuracy (WBL%, mMPTA, HKA, PTSA) and conversion to a unified index; (iv) full‐text articles; and (v) text in English or Chinese language.

The exclusion criteria were as follows: (i) reviews, case reports, or conference abstracts; (ii) duplicated data publications from the same authors and studies; (iii) results with insufficient or incorrect data; and (iv) other exclusion case reports and small case series (n < 10).

### Methodological Quality Assessment

The final search results included observational studies but no randomized controlled trials. To assess the methodological quality of the nonrandomized surgical studies, we made use of a validated instrument called “MINORS” (Methodological Index for Nonrandomized Studies)^19^ and “NOS” (Newcastle–Ottwa Scale).^20^ Each of the methodological items of the MINORS index was subsequently graded on a three‐point scale ranging from 0 to 2. The MINORS index contains eight items for noncomparative studies and 12 items for comparative studies, with a highest overall score of 16 for the former and 24 for the latter. The literature was regarded as of low quality when the score of the NOS was less than 5 points. The scoring of each article is shown in Table 1. Each article was scored by two authors (R. P. and C. X.). A third author resolved disagreements by adjudication (Z. J.).

**TABLE 1:** Demographic characteristics of the PSI technique studies

Authors	Study design	Age (mean)	Group	Number	Gender		Minors	NOS
					M	F		
Munier 2017^7^	POS	46	‐	10	10		10	6
Jacquet 2020^8^	PRCS	44	‐	71	32	39	12	6
Chaouche 2019^9^	PRCS	44	‐	100	59	41	12	6
Tardy 2020^10^	PCS	51	conventional	126	44	17	16	8
			navigation		21	5		
			PSI		35	4		
Mao 2020^15^	PCS	44	PSI	18	4	14	18	8
		42	conventional	19	5	14		
Kim 2018^16^	RCS	55	conventional	20	4	16	16	8
			PSI	20	3	17		
Fucentese 2020^21^	PRCS	45	‐	23	16	7	10	5
Gao 2021^22^	ROS	61	‐	14	5	9	10	6
Genechten 2020^23^	POS	47	‐	10	6	4	12	6
Predescu 2021^24^	ROS	<60	‐	25	‐	‐	12	6
Yang 2018^25^	PRCS	67	‐	10	4	6	10	6

Abbreviations: F, females; M, males; Minors, Methodological Index for Nonrandomized Studies; NOS, Newcastle‐Ottawa Scale; PCS, prospective comparative study; POS, prospective observational study; PRCS, prospective cohort study; PSI, patient‐specific instrumentation; RCS, retrospective comparative study; ROS, retrospective observational study.

### Outcome Assessment

The desired result of HTO is target limb alignment correction. There is, however, no consensus on the ideal way to evaluate the correction of limb alignment. The target of coronal correction after open‐wedge HTO has been evaluated with angular correction referring to the HKA angle and mMPTA and in terms of the WBL%. The target of sagittal correction is typically evaluated using PTSA. In some studies, the difference in the preoperatively planned and postoperatively achieved correction (ΔHKA, ΔmMPTA, ΔPTSA) was defined as accuracy.^8^, ^9^, ^10^, ^15^, ^21^ All indices were measured using 2D imaging or 3D CT scans.

Related indicators of other secondary outcomes, such as operative time, fluoroscopic number, and complications, were also noted.

### Data Extraction and Statistical Analysis

Two authors (X. Z. and W. S.) extracted the following data from the selected articles: study design, patient demographics, the preoperative and postoperative measurement indices (WBL%, HKA, MPTA, PTSA) or their pre‐ to postoperative difference (ΔHKA, ΔmMPTA, ΔPTSA) and the designed target value in each study. At the same time, the following data were also extracted and addressed in the discussion: complications, follow‐up duration, functional outcomes, surgery time, and fluoroscopy time/number. It was important to convert the data from the various indices to unify them.

Review Manager statistical software from the Cochrane Collaboration was used to conduct a meta‐analysis (RevMan, 5.4). Dichotomous variables are represented as the risk ratio (RR) and odds ratio (OR), and continuous variables are summarized as the mean difference (MD). The *p* value for statistical significance was set to 0.05, and the mean difference (MD) was reported using the 95% confidence interval (95% CI). Statistical heterogeneity was evaluated with I‐square (I^2^) tests. When *p* < 0.1 or I^2^ > 40%, the heterogeneity was considered statistically significant. When the heterogeneity was considerable (I^2^ > 40%), a random effects model was utilized; otherwise, a fixed effects model was used.

## Results

### Search Results

**Fig. 1:**
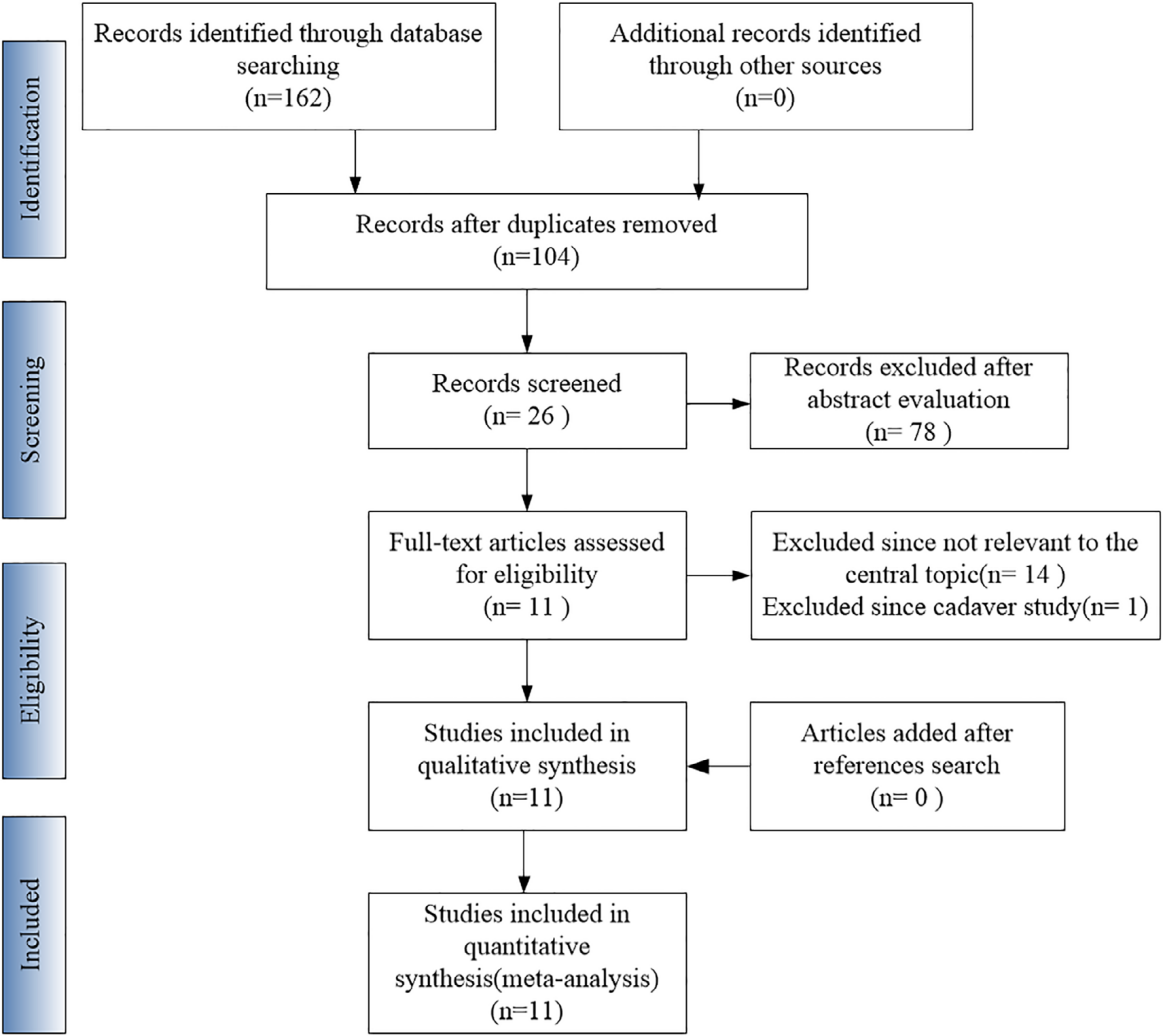
Flow chart showing identification and selection of cases

A total of 162 potential pertinent studies were preliminarily detected. After removing duplicates (n = 58), the remaining studies (n = 104) were assessed through a review of the titles and abstracts. Seventy‐eight studies were excluded, leaving 26 eligible studies. After assessing the full texts of the 26 articles, 14 were excluded because they were not relevant to the central topic (non‐HTO studies, nonclinical research, basic theoretical research, too few cases [n < 10]), and 1 was excluded because it was a cadaver study.^26^ Subsequently, we conducted a manual search of all included studies' reference lists. Similarly, as mentioned above, articles that deviated from the central topic or had only a few cases were excluded. By the end of this step, 11 articles met the inclusion criteria. The flow chart for the study selection process is shown in Figure 1.

### Methodological Quality Assessment

An evaluation of the included articles was conducted using the MINORS and the NOS. The studies had a mean MINORS score of 12.5, with a range of 10–18, which indicated that the included articles were of higher quality; consistent results were obtained when assessed by applying the NOS, with the score ranging from 5 to 8 (Table 1). Notably, six of the 11 selected articles were involved in a meta‐analysis published in 2020.^11^ The quality assessment of the studies in that literature and our work was very consistent.

### Patients and Study Characteristics

The demographic characteristics of the study cohorts are summarized in Table 1. The total number of patients included in the 11 studies was 466. Two of these studies compared conventional and PSI HTO,^15^, ^16^ and one compared all three preoperative planning methods.^10^ The literature included three comparative studies, three observational studies, five cohorts but no randomized controlled trials.

### Primary Outcome: Accuracy of Postoperative Alignment for HTO

The included studies reported data with different evaluation indices. Tables 2 and 3 display the results of the evaluation indices.

**TABLE 2:** The accuracy of PSI for HTO

Authors	Groups	Accuracy		
		△HKA	△mMPTA	△PTSA
Jacquet 2020^8^	‐	1.0 ± 1.0	0.5 ± 0.6	0.4 ± 0.8
Chaouche 2019^9^	‐	1 ± 0.9	0.5 ± 0.6	0.4 ± 0.8
Tardy 2020^10^	conventional	1.1 ± 3	‐	‐
	navigation	2.1 ± 2.6	‐	‐
	PSI	0.3 ± 3.1	‐	‐
Mao 2020^15^	PSI	0.2 ± 0.6	0.1 ± 0.4	‐
	conventional	1.2 ± 1.4	2.2 ± 1.8	‐
Fucentese 2020^21^	‐	‐	0.8 ± 1.5	1.7 ± 2.2

Abbreviations: △, the difference in the preoperative planned and postoperative achieved correction; HKA, hip‐knee‐ankle; mMPTA, mechanical medial proximal tibia angle; PSI, patient‐specific instrumentation; PTSA, posterior tibial slope angle.

**TABLE 3:** The values of WBL%, HKA, MPTA, PTSA in preoperative measurements, designed target, and postoperative measurements

Authors	Groups	Preoperative measurements values				Designed target values		Postoperative measurements values			
		WBL%	HKA(°)	mMPTA(°)	PTSA(°)	HKA(°)	mMPTA(°)	WBL%	HKA(°)	mMPTA(°)	PTSA(°)
Munier 2017^7^	‐	‐	172.3 ± 3.5	85.5 ± 1.4	7.1 ± 5.6	182.4 ± 0.8		‐	182.4 ± 1.2	94.7 ± 3.0	6.4 ± 3.7
Chaouche 2019^9^	‐	‐	171.6 ± 2.8	79.8 ± 3.1	9.2 ± 3	‐	‐	‐	182.1 ± 1.3	90.6 ± 1.8	7.6 ± 2
Tardy 2020^10^	Conventional	‐	174.3 ± 3.1	84.9 ± 3.9	‐	182.4 ± 1.7	‐	‐	181.5 ± 3.7	89.4 ± 12.1	‐
	Navigation	‐	174.1 ± 2.9	85.5 ± 2.3	‐	183.3 ± 0.5	‐	‐	181.1 ± 2.6	91.6 ± 2.3	‐
	PSI	‐	172.9 ± 2.8	83.9 ± 3.1	‐	181.1 ± 2.6	‐	‐	180.8 ± 2.8	92 ± 3.1	‐
Mao 2020^15^	PSI	‐	172.2 ± 1.7	86.3 ± 2.28	‐	180.5 ± 0.91	91.3 ± 0.87	‐	180.7 ± 0.7	91.2 ± 0.65	‐
	Conventional	‐	173.3 ± 1.7	83.4 ± 2.15	‐	‐	‐	‐	179.7 ± 1.8	89.3 ± 2.13	‐
Kim 2018^16^	Conventional	19.4 ± 12.2	173.1 ± 3.1	‐	9.9 ± 3.0	‐	‐	61.3 ± 8.1	183.1 ± 2.3	‐	10.5 ± 2.6
	PSI	21.2 ± 11.8	172.6 ± 2.7	‐	8.6 ± 3.3	‐	‐	61.6 ± 3.3	183.5 ± 1.2	‐	8.9 ± 3.1
Fucentese 2020^21^	‐	‐	172.9 ± 3.0	‐	6.4 ± 3.0	‐	‐	‐	181.7 ± 2.5		8.1 ± 3.5
Gao 2021^22^	‐	28.7 ± 8.4	174.5 ± 2.1	83.3 ± 1.5	10.0 ± 2.9	182.6 ± 0.6	90.5 ± 0.9	59.5 ± 2.5	182.1 ± 1.0	90.3 ± 1.7	10.8 ± 3.4
Genechten 2020^23^	‐	‐	‐	‐	5.9 ± 2.8	181.0 ± 1.2	91.4 ± 3.3	‐	180.5 ± 1.3	91.7 ± 2.4	4.4 ± 2.6
Predescu 2021^24^	‐	‐	167.0 ± 2.5	82.5 ± 1.37	7.9 ± 2.52	‐	‐	‐	182.2 ± 1.15	92.8 ± 0.6	6.5 ± 2.18
Yang 2018^25^	‐	21.8 ± 7.6	‐	‐	9.9 ± 0.4		‐	60.2 ± 2.6	‐	‐	10.1 ± 0.3

Abbreviations: HKA: hip‐knee‐ankle; mMPTA: mechanical medial proximal tibia angle; PSI, patient‐specific instrumentation; PTSA: posterior tibial slope angle; WBL: weight bearing line.

**Fig. 2:**
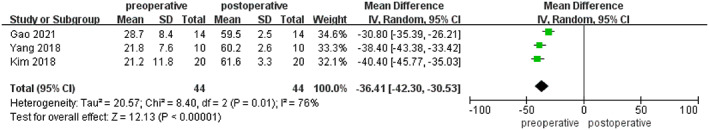
Comparison of WBL% in the pre‐ and postoperative measurements

There were only three articles that reported WBL%. In these three studies, significant differences in WBL% were detected in the preoperative and postoperative measurement groups (MD = −36.41; 95% CI –42.30 to −30.53; *p* < 0.00001; Figure 2).

**Fig. 3:**
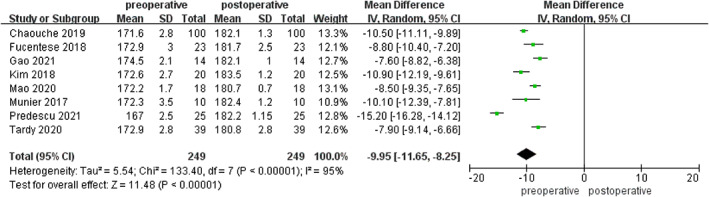
Comparison of HKA in the pre‐ and postoperative measurements

**Fig. 4:**
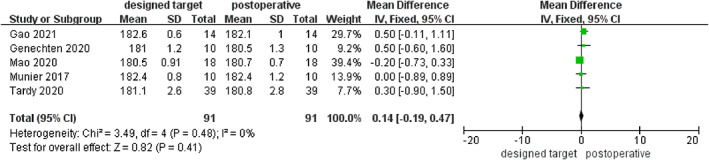
Comparison of HKA in the designed target values and postoperative measurements

**Fig. 5:**
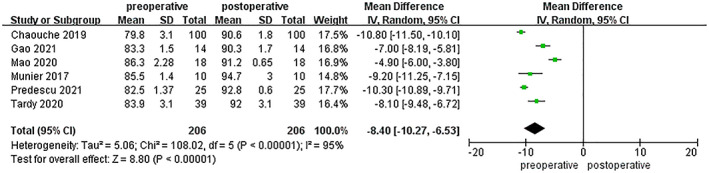
Comparison of mMPTA in the pre‐ and postoperative measurements

**Fig. 6:**
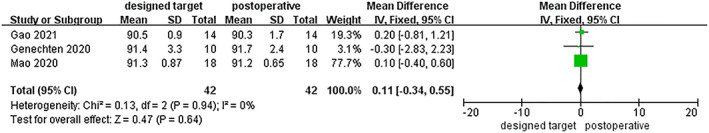
Comparison of mMPTA in the designed target and postoperative measurements

The indices of coronal correction also included HKA and mMPTA. Significant differences were detected in the comparison between the preoperative and postoperative HKA in the eight included studies, which included a total of 249 patients (MD = −9.95; 95% CI −11.65 to −8.25; *p* < 0.00001; Figure 3).There were no major differences discovered in the comparison between the designed target and postoperative HKA (MD = 0.14; 95% CI −0.19 to 0.47; *p* = 0.41; Figure 4). Data on mMPTA were available from six studies, which included a total of 206 patients. These studies had significant heterogeneity (I^2^ = 95%), and analysis using a random effects model revealed significant between‐group differences in preoperative and postoperative measurements (MD = –8.40; 95% CI −10.27 to −6.53; *p* < 0.00001; Figure 5), whereas no significance was found between the designed target and postoperative mMPTA (MD = 0.11; 95% CI −0.34 to 0.55; *p* = 0.64; Figure 6). The above shows that a PSI could guarantee the accuracy of coronal correction.

**Fig. 7:**
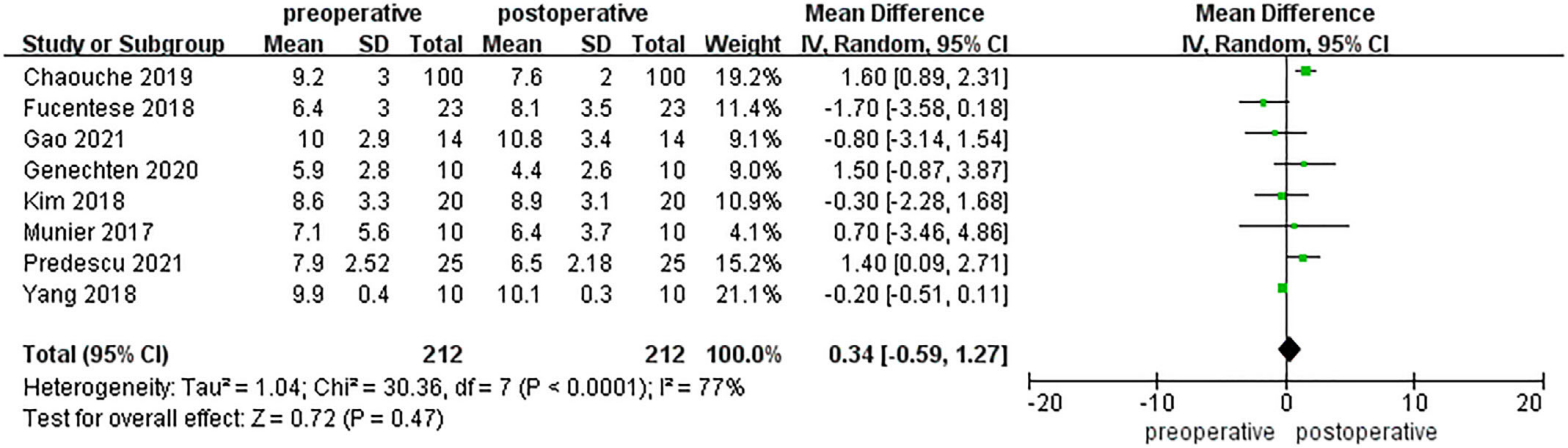
Comparison of PTSA in the pre‐ and postoperative measurements

PTS was described in eight investigations involving 212 patients, although there was no significant difference between the pre‐and postoperative groups (MD = 0.34; 95% CI −0.59 to 1.27; *p* = 0.47; Figure 7). The heterogeneity was high (I^2^ = 77%), but it decreased to I^2^ = 43% when the study by Chaouche *et al*. was excluded. A PSI was thus also sufficiently accurate to maintain sagittal correction.

### Secondary Outcome

Several articles reported complications, follow‐up time durations, functional outcomes, surgery time, fluoroscopic number, and learning curves (Table 4). Eight of the 11 included articles explicitly mentioned complications, although two of these reported that no complications occurred during the study.^7^, ^22^ Another study reported that major complications included Takeuchi fractures, wound infections, and bone nonunion.^9^ No major complications were mentioned aside from those noted above. Four studies reported the incidence of minor complications, such as Takeuchi type 1 benign lateral hinge fractures and postoperative hematoma. The length of time between follow‐ups varied throughout the included papers, ranging from 3 to 24 months, with an average of 12 months. Jacquet *et al*. also introduced functional outcome (assessed by KOOS), mean surgery time (26.3 ± 8.8 minutes), fluoroscopic image number (4.3 ± 1.2), and learning curve (assessed by CUSUM analysis).^8^ The other two scores (IKDC and Lysholm score) were used to assess the subjective knee function of the patients by Mao *et al*..^13^ As shown in Table 4, three other studies described the duration of surgery, and two of them also referred to the number of fluoroscopies.^15^, ^24^, ^25^

**TABLE 4:** The second outcomes assessment

Authors	Group	FU(M)	Complications	Functional outcomes	Surgery time(min)	Fluoroscopic number
Munier 2017^7^	‐	12	0/10	‐	‐	‐
Jacquet 2020^8^	‐	12	‐	KOOS	26.3 ± 8.8	4.3 ± 1.2
Chaouche 2019^9^	‐	24	32/100	KOOS; UCLA	‐	‐
Tardy 2020^10^	conventional	12	‐	‐	‐	‐
	navigation		‐	‐	‐	‐
	PSI		‐	‐	‐	‐
Mao 2020^15^	PSI	12	2/18	IKDC; Lysholm	37.8 ± 7.14	1.3 ± 0.12
	conventional		6/19		54.6 ± 11.72	4.1 ± 0.57
Kim 2018^16^	conventional	12	‐	‐	‐	‐
	PSI		‐	‐	‐	‐
Fucentese 2020^21^	‐	3	3/23	‐	‐	‐
Gao 2021^22^	‐	14.8	0/14	HSS	‐	‐
Genechten 2020^23^	‐	12	2/10	‐	‐	‐
Predescu 2021^24^	‐	12	2	‐	40	10
Yang 2018^25^	‐	6	‐	‐	30	‐

Abbreviations: FU, follow‐up time duration; HSS, the Hospital for Special Surgery; IKDC, the International Knee Documentation Committee; KOOS, Knee injury and Osteoarthritis Outcome Score; Lysholm, Lysholm score; M, month; UCLA, University of California Los Angeles activity scale.

## Discussion

The purpose of our study was to assess the accuracy of correction in HTO with PSI and to explore the assessment indices of correction in HTO.

### Correction Assessment Indices

The selected studies reported sagittal correction with PTS and coronal correction with WBL%, HKA angle, and mMPTA to evaluate the accuracy of surgery. The weight bearing line was detected using preoperative weight‐bearing coronal plane radiography of the whole leg. The WBL% is defined as the ratio of the horizontal distance from the intersection point (between the weight bearing line and the joint line of the proximal tibia) to the medial edge of the tibial plateau divided by the entire width of the tibial plateau.^16^ The planned correction of the WBL was made if the line passed through Fujisawa's point, which suggests a WBL% of 62.5%.^21^, ^27^ Fujisawa *et al*. determined that the target range of WBL should be 65%–70% of the tibial width.^27^ This corresponds to a postoperative HKA of 183°–185°.^11^ In recent years, according to several researchers, changing the WBL to 50%–55% can improve the clinical outcomes.^28^ While some authors discuss the variability of the postoperative WBL%,^29^ Genechten *et al*. achieved a reliable planning angle using mMPTA with 3D software during HTO.^23^ To maintain the knee biomechanics, it is crucial to control the sagittal plane of correction.^30^, ^31^ Sagittal correction is typically evaluated using the posterior tibial slope in the relevant literature.^20^, ^21^, ^22^, ^23^, ^26^, ^27^, ^28^, ^29^, ^30^, ^31^, ^32^, ^33^, ^34^ PTS should be kept unchanged or as close to unchanged as possible pre‐and postoperatively. Due to the lack of comparison between these indices, there is no consensus on which index is the best ideal correction index after medial open‐wedge HTO.^11^ The meta‐analysis article suggests that subsequent studies use the above four measurement indicators to evaluate the accuracy of PSI HTO to facilitate the comparison between different studies.

### Advantages and Accuracy of PSI Applied to HTO

PSIs have been widely recognized in reducing the surgeon's learning curve and the need for intraoperative fluoroscopy, shortening the surgical duration, and improving surgical correction accuracy. Jacquet *et al*. reported that the use of PSIs had no effect on the accuracy of correction or functional outcomes and could shorten the learning curve.^8^ The study claimed that the mean number of images taken was 4 ± 1 while using a PSI, which reduced unnecessary radiation exposure for the patients and the operative team.^8^ Another study demonstrated that the number of fluoroscopic images taken decreased from an average of 55 using conventional methods to eight using PSI.^35^ The number of intraoperative fluoroscopy images was only 1.3 ± 0.12 times in the study of Mao *et al*..^15^ Some of the selected studies also demonstrated that the use of PSIs is associated with a reduction in surgical time. Furthermore, CT scanning and 3D modeling aid in the visualization of patient anatomy, process comprehension, and the accuracy of the planned correction.^6^, ^19^ PSI is applicable in highly complex surgical interventions, especially those that require multiplanar or rotational osteotomies.^17^ This is consistent with Van Genechten *et al*., who proposed that the availability of PSI contributes to more accurate and satisfactory results, especially in cases of double‐level osteotomies and multiplanar deformities. Using the KOOS and UCLA activity scale, PSIs for open‐wedge HTO have also been linked to better socio‐professional results, including a faster return to work and more physical activity, according to Chaouche *et al*..^9^ Knee function after surgery was evaluated in several other studies by the HSS and other scales, and the results showed that all of their scores were significantly improved.^8^, ^9^, ^15^, ^22^

Generally, the use of PSIs in HTO can lead to accurate corrections. When compared to traditional surgical techniques, a meta‐analysis published in 2020, analyzing 18 studies focusing on CAS and 10 on PSI, found that adopting CAS resulted in a significantly lower outlier rate and a non‐significantly higher accuracy.^11^ However, the results of PSI use are still inconclusive.^11^ Six of the 11 studies included in our meta‐analysis were included in the aforementioned study. We also analyzed five additional studies published more recently. All 11 included articles that determined that the use of PSIs could increase the correction accuracy in HTO as a whole [7, 8, 9, 10, 15, 16, 22, 23, 27, 35, 36]. WBL%, HKA, and mMPTA acted as evaluation indices for coronal correction. There were significant differences between the pre‐and postoperative measurements of these indicators, but the heterogeneity was very high (WBLI^2^ = 76%; both HKA and mMPTAI^2^ = 95%). There was no significant difference between the design target value and the postoperative measured value, and the heterogeneity was low (both HKA and mMPTAI^2^ = 0%). We attempted a subgroup analysis but found no significant decrease in heterogeneity when studies were removed. This result suggested that the high heterogeneity may be from the studies themselves. The eight studies that looked at PTS as an evaluation index for sagittal correction found no significant difference between the pre‐and post‐surgical groups. It seems PSIs could maintain the PTS in HTO.

### Controversial Issue of PSI Application

Nonetheless, studies on the application of PSI are controversial for the following reasons. Although we found that the number of fluoroscopic images taken did decrease, some authors note that preoperative CT scans performed for planning also cause unnecessary radiation exposure.^9^ However, this is substantially lower (0.16 mSv) than the radiation from intraoperative fluoroscopy exposure (approximately 20–200 mSv per minute).^25^ As discussed previously, major complications in the selected studies included Takeuchi fractures, wound infections, and bone nonunion.^9^ Other complications mentioned aside from the above were classified as minor events.^7^, ^21^ According to Chaouche *et al*., 24% of fractures were detected with a CT scan, which is consistent with other publications.^9^, ^36^, ^37^ However, the incidence of overall complications is still unknown. Most studies that assess the accuracy of PSI use relied on the use of prominent bony landmarks. Fucentese *et al*. found that the inaccurate placement of PSIs and the initial learning curve may result in decreased surgical accuracy.^21^ PSIs may themselves also be inaccurate. A study using computer simulation determined that improvements in PSI technology, such as guides with additional stabilizing hooks, would lessen the chance of incorrect screw installation. However, this study also found that an inaccurately placed guide within the margin of error had no effect on the postoperative mechanical axis.^38^ In general, if only accurate correction of the osteotomy is desired, universal cutting guides and more precise surgical instruments can play a helpful role.

There are some notable limitations in our study. There is a dearth of relevant literature on the use of PSIs in open‐wedge HTO. Three articles were purposely excluded due to limited case numbers, incomplete data or preoperative planning that did not involve a PSI. It is also possible that some existing articles were missed in our preliminary search. Next, we found only one ongoing RCT study on ClinicalTrials.gov and no other randomized controlled trials. Most of the included articles were observational comparative studies, which have a low level of evidence.

Our researchers believe that the application of PSIs is more suitable for surgeries necessitating a more complex osteotomy but not necessary in a typical HTO.

### Conclusions

In the preoperative planning of open‐wedge HTO, WBL%, HKA angle, and mMPTA best represent coronal correction, whereas sagittal correction is best represented by PTS. The optimal correction index could not be determined. We concluded that the use of PSIs could increase the accuracy of the correction in HTO, especially in complex cases. However, due to the lack of clinical randomized controlled trials, the accuracy of the correction between conventional and CAS‐ and PSI‐based procedures is still unknown. Last, our study determined that PSIs are accurate, but their application is not necessary in typical HTO.

## Authorship Contribution Statement

R. P. and Z. J. contributed equally to this work and should be considered co‐first authors. They conceived and identified the topic of the study. R. P., C. X., W. S., and X. Z. reviewed and edited the first draft of this work. Z. J. and X. W. extracted the data and completed the statistical analysis. H. L., F. S., and I. B. contributed to data analysis and improved the manuscript. The language of the revised manuscript has been refined and polished by D. B. All authors have given approval to the final version of the manuscript to be published.

## Conflict of Interest

All authors certify that he or she has no funding or commercial associations that might pose a conflict of interest in connection with the submitted article.
